# Social goals and coping with interpersonal stress in adolescence

**DOI:** 10.3389/fpsyg.2026.1753006

**Published:** 2026-07-08

**Authors:** Yuji Kuroda

**Affiliations:** Institute of Psychology, University of Tsukuba, Tsukuba, Japan

**Keywords:** coping, friendship, interpersonal stress, social achievement goals, social goal orientation

## Abstract

**Introduction:**

Prior studies have found that three types of social goals—social learning, social performance-avoidance, and social performance-approach goals—predict stress coping among adolescents. However, prior findings are limited by the range of stress contexts and coping forms examined. Furthermore, questions remain regarding how social performance-approach and performance-avoidance goals differ in predicting coping. To address these gaps, this study examined whether the three social goals differentially predict engagement, disengagement, and mixed forms of coping in the context of friend-related stress—an important but unexplored stress context and set of coping forms.

**Method:**

Participants were 137 students (67 girls) recruited from a public middle school in Japan. Data were collected using a cross-sectional design. A path analysis was conducted to examine the hypothesized associations.

**Results:**

As hypothesized, social learning goals were associated with relationship-rebuilding and positive reappraisal coping, a form of engagement (adaptive) coping; social performance-avoidance goals were associated with relationship-withdrawal coping, a form of disengagement (maladaptive) coping; and social performance-approach goals were associated with relationship-changing coping, a mixed form of engagement and disengagement (adaptive and maladaptive) coping. Although not initially hypothesized, social performance-approach goals were also associated with relationship-rebuilding and positive reappraisal coping.

**Discussion and conclusion:**

These findings support the predictive utility of the trichotomous framework of social goal orientations across stress contexts and coping forms. Furthermore, our findings support the discriminative utility of this framework by highlighting the contrasting roles of the three goals in coping. Notably, whereas social performance-avoidance goals may serve maladaptive functions in coping, social performance-approach goals seem to serve both maladaptive and adaptive functions.

## 1 Introduction

Friendships and peer relationships in school contexts have critical implications for social, emotional, and academic adjustment during adolescence (Ryan and Ladd, 2012; Wentzel et al., 2004). Interpersonal stress arising in these relationships, such as peer rejection, criticism, and conflict, is a potential risk factor for internalizing problems among adolescents (Kong et al., 2022; Platt et al., 2013). Therefore, it is essential to elucidate why some adolescents are more vulnerable to such stress whereas others respond more resiliently.

Several theoretical perspectives help explain these individual differences, including cognitive (e.g., self-schema and social information processing), interpersonal (e.g., attachment), and motivational (e.g., social goal orientation) perspectives. Among these, we focus on social goal orientation theory. This theory provides a unified, parsimonious framework for understanding both adaptive and maladaptive responses to social failures and interpersonal challenges (Dweck, 1996; Dweck and Leggett, 1988). The theory is particularly well suited to research on vulnerability and resilience to interpersonal stress during adolescence, a developmental period in which individuals become increasingly concerned about peer evaluations while learning how to develop peer relationships (Blakemore and Mills, 2014; Brown and Larson, 2009; Westenberg et al., 2004). Given this theoretical relevance and suitability, the present study sought to elucidate how social goals predict individual differences in coping responses to interpersonal stress, thereby contributing to a motivational understanding of adolescents’ vulnerability and resilience to such stress.

Social goal orientation theory (Dweck, 1996; Dweck and Leggett, 1988; Erdley et al., 1997) explains how social goals shape mastery- versus helpless-oriented responses to social failures and challenges. Social goals generally refer to what individuals focus on and try to accomplish when interacting with their peers (Ryan and Shim, 2006; Shim et al., 2013).^1^ The theory postulates two fundamental classes of social goals to explain the above contrasting responses parsimoniously. The first type is social learning goals, which focus on growth and mastery through social interactions. These goals include developing one’s social attributes, gaining social knowledge, and cultivating social relationships. The second type is social performance goals, which focus on validating and judging one’s social attributes through social interactions.^2^ These goals include seeking positive evaluations and avoiding negative evaluations. Social learning goals encourage individuals to view social challenges as opportunities for growth and development, and thus facilitate mastery-oriented responses. In contrast, social performance goals frame these situations as evaluative tests, leading to helpless-oriented responses.

Subsequent studies (Horst et al., 2007; Kuroda and Sakurai, 2001; Rudolph et al., 2011; Ryan et al., 2004; Ryan and Shim, 2006) have refined this perspective by dividing social performance goals into approach and avoidance dimensions that are fundamental to motivation, thereby yielding a three-goal classification of social goal orientations (hereafter, the trichotomous framework of social goal orientations). Social performance-approach goals aim to gain positive evaluations of one’s social attributes, whereas social performance-avoidance goals focus on avoiding negative judgments of them. Studies have demonstrated that these goals have different associations with psychological adjustment outcomes (for reviews, see Dawes, 2017; Ryan et al., 2012; Shim and Ryan, 2019). Specifically, social learning goals typically show positive associations with psychological adjustment, whereas social performance-avoidance goals show negative associations. In contrast, social performance-approach goals generally exhibit null associations with such outcomes. Particularly relevant to this study are Kuroda and Sakurai’s (2011) findings that the three social goals showed different interaction patterns with interpersonal stress in predicting depressive symptoms. Specifically, (a) social learning goals buffered the effects of interpersonal stress, thereby protecting against depressive symptoms; (b) social performance-avoidance goals exacerbated the effects of interpersonal stress, thereby increasing symptoms; and (c) social performance-approach goals neither buffered nor exacerbated the effects of interpersonal stress. This finding suggests that social learning goals may predict adaptive coping responses to interpersonal stress, whereas social performance-avoidance goals may predict maladaptive coping responses. In contrast, social performance-approach goals may play a more complex role and potentially predict both adaptive and maladaptive coping responses to interpersonal stress.

However, despite this body of empirical evidence, few studies have investigated how the three social goals predict specific responses to interpersonal stress among adolescents. Two notable exceptions are the studies by Shin and Ryan (2012) and Michou et al. (2016). These studies investigated how the three goals predict coping styles, which refer to relatively stable individual differences in the tendency to use particular coping strategies within a given stress domain (Carver and Connor-Smith, 2010; Moos and Holahan, 2003).

Shin and Ryan (2012) examined coping with peer-related problems occurring outside friendships, focusing on students’ use of friends as a coping resource when encountering difficulties with their peers at school. They classified coping strategies into mastery (efforts to understand and resolve the problem; e.g., discussing solutions with friends), nonchalance (efforts to downplay the problem; e.g., telling friends that the issue is not significant), and avoidance (efforts to escape the problem; e.g., concealing the problem from friends). They found positive associations between social development goals and mastery coping, social demonstration-approach goals and nonchalant coping, and social demonstration-avoid goals and avoidance coping.

Michou et al. (2016) examined coping with general stress rather than interpersonal stress, including coping strategies such as adaptive styles (e.g., active coping, planning, and positive reframing) and defensive styles (e.g., humor, denial, and emotional venting). They found that adolescents pursuing both social development and demonstration-approach goals scored higher on defensive coping strategies than those pursuing social development goals only. This finding suggests that social demonstration-approach goals facilitate defensive coping strategies.

Taken together, these studies suggest that social development goals (corresponding to social learning goals) are associated with adaptive coping strategies, whereas social demonstration-avoid and demonstration-approach goals (corresponding to social performance-avoidance and performance-approach goals, respectively) are associated with maladaptive but distinct coping styles.

Although these pioneering studies have made valuable contributions to the literature, three important gaps remain. First, the scope of interpersonal stress contexts remains limited to peer-related problems occurring outside friendships (Shin and Ryan, 2012) and stress in a general rather than relationship-specific domain (Michou et al., 2016). In this study, we focused on friend-related stress (i.e., stress arising in relationships with friends). Examining coping in this context is developmentally and clinically critical, because friendships become more salient during adolescence, and friend-related stress jeopardizes adolescents’ mental health (Benner et al., 2020; Kong et al., 2022).

Second, to establish a more integrative understanding of social goals and coping, it is necessary to move beyond the coping classifications used in prior social goal research and connect this line of research to the broader theoretical and empirical traditions of adolescent coping research. To address this issue, this study focused on engagement and disengagement forms of coping. Although there are various coping strategies and debates over their categorization (e.g., Skinner et al., 2003), Connor-Smith et al. (2000) used adolescent samples to provide robust factor-analytic support for the distinction between engagement and disengagement. Furthermore, a meta-analysis by Compas et al. (2017) demonstrated that this framework has discriminant validity in relation to mental health indicators, showing that engagement forms are associated with better mental health, whereas disengagement forms are associated with poorer outcomes. Accordingly, empirical studies on adolescent coping have examined these forms of coping (e.g., Erath and Pettit, 2021; Zimmer-Gembeck and Skinner, 2024).

Engagement coping involves approach responses directed toward a stressor or one’s emotions or thoughts, including primary control or assimilative coping (i.e., changing the stressful situation) and secondary control or accommodative coping (i.e., adapting to the situation) (Connor-Smith and Flachsbart, 2007; Connor-Smith et al., 2000). In contrast, disengagement coping involves responses oriented away from a stressor or one’s emotions or thoughts, including avoidance (Connor-Smith et al., 2000). Considering the theoretical predictions of this study (see the hypotheses below), we examined three specific strategies. First, for engagement coping, we focused on efforts to maintain, repair, or improve relationships with friends (primary control engagement) and to view stress as an opportunity for growth and development (secondary control engagement). Hereafter, we refer to these strategies as *relationship-rebuilding and positive reappraisal coping*. Second, for disengagement coping, we focused on efforts to withdraw from friendships and peer relationships completely. We refer to these strategies as *relationship-withdrawal coping*. Third, for mixed forms of disengagement and engagement coping, we examined efforts to avoid stressful friends while actively seeking new friends or maintaining other existing friendships. The former avoidance effort reflects a disengagement form. In contrast, the latter type of active effort reflects an accommodative form of engagement coping because it involves an attempt to adapt to the social environment by seeking or maintaining other compatible relationships. We refer to these mixed strategies as *relationship-changing coping*.

Third, questions remain regarding how social performance-avoidance and social performance-approach goals differ in their roles in coping with stress. Shin and Ryan (2012) and Michou et al. (2016) found that social performance-approach goals play maladaptive roles in coping, suggesting that these goals may function similarly to social performance-avoidance goals. However, as discussed above, prior studies have demonstrated that social performance-approach goals neither buffer nor exacerbate the effects of interpersonal stress (Kuroda and Sakurai, 2011) and generally exhibit null associations with psychological adjustment (Shim and Ryan, 2019). This raises the possibility that social performance-approach goals may serve not only maladaptive but also adaptive functions in the coping process. We tested this possibility by examining the association between these goals and relationship-changing coping.

Addressing these three gaps is necessary to advance the literature in theoretically significant ways. By examining different coping forms in different stress contexts, we can evaluate the broader predictive utility of social goal orientation theory for stress coping. Furthermore, by examining the functional differences between social performance-approach and performance-avoidance goals, we can evaluate the discriminative utility of the trichotomous framework of social goal orientations.

Therefore, this study aimed to examine how the three social goals were associated with engagement, disengagement, and mixed coping responses to friend-related stress. In this context, the following hypotheses were tested.

Social learning goals focus on personal growth and relational development. Adolescents with such goals would utilize engagement coping with friend-related stress because such stress facilitates rather than hinders their pursuit of these goals. Specifically, they would make efforts to maintain, repair, or improve relationships with friends (e.g., better understanding friends) and view stress as an opportunity for growth and development. Thus, we formulated Hypothesis 1 as follows: Social learning goals would be positively associated with relationship-rebuilding and positive reappraisal coping.

Social performance-avoidance goals aim to avoid negative evaluations of one’s social attributes, making individuals focus on the possibility of receiving negative feedback from others. Adolescents with these goals would adopt disengagement coping with friend-related stress. Specifically, when encountering negative behavior by a friend (e.g., teasing, criticism, or ignoring), they would withdraw from that friend because such behavior signals the very evaluations they seek to avoid, and therefore elicits feelings of threat and fear. They would further avoid interactions with other friends and peers, as the elicited threat and fear lead them to focus exclusively on the possibility of being negatively evaluated again in the future. As such, adolescents with social performance-avoidance goals would completely withdraw from friendships and peer relationships. Based on this theoretical reasoning, Hypothesis 2 was formulated as follows: Social performance-avoidance goals would be positively associated with relationship-withdrawal coping.

Social performance-approach goals aim to gain positive evaluations of one’s social attributes, driving individuals to focus on the possibility of receiving positive feedback from others. Adolescents with these goals would use mixed coping strategies that encompass both maladaptive (disengagement) and adaptive (engagement) responses. Specifically, when encountering negative behavior by a friend, they would avoid that friend (disengagement) because the behavior contradicts the positive feedback they desire, and therefore causes frustration. However, unlike adolescents with social performance-avoidance goals, those with social performance-approach goals would strive to form new friendships with other peers or to maintain other existing friendships (engagement), as the elicited frustration leads them to seek positive feedback and restore positive evaluations. Based on this theoretical reasoning, Hypothesis 3 was formulated as follows: Social performance-approach goals would be positively associated with relationship-changing coping.

In testing these hypotheses, we considered the following two points. First, given that prior research found sex differences in social goal orientations (Kuroda and Sakurai, 2011), we controlled for sex in the analysis of the hypothesized model. Second, regarding the age group in our study, we focused on early adolescence from clinical and developmental perspectives. Clinically, early adolescence is a critical period characterized by marked increases in internalizing symptoms. Developmentally, it is a stage during which substantial psychological and social development is underway, making early adolescence an opportune time for prevention and intervention. Given these considerations, this period is essential for identifying risk and protective factors and cultivating resilience while reducing vulnerability (Kuroda, 2023). We selected seventh and eighth graders for this study because, in Japan, seventh grade is the first year of junior high school and marks a major transition from elementary school. During this period, students experience substantial shifts in interpersonal dynamics and must navigate increased peer-related stress and conflict.

## 2 Materials and methods

### 2.1 Participants and study design

The participants were seventh (12–13 years old) and eighth (13–14 years old) graders recruited from a public junior high school in Japan using convenience sampling. Of the 166 students who participated in the study, 27 were excluded because they provided incomplete data, and two were excluded because they gave the same response to all items. Thus, the final analytic sample consisted of 137 students (67 girls; 83 seventh graders). The study design was cross-sectional, and data were collected using self-report questionnaires.

### 2.2 Measures

#### 2.2.1 Social goal orientations

We used the Social Goal Orientations Scale (SGOS; Kuroda and Sakurai, 2001), which comprises three subscales measuring social learning goals, social performance-approach goals, and social performance-avoidance goals. The social learning goal subscale consists of ten items assessing goals of developing oneself through interpersonal experiences (e.g., “I want to get to know peers who think differently from me and talk with them”). The social performance-approach goal subscale consists of seven items assessing goals of obtaining positive evaluations of one’s social attributes (e.g., “I want to give my friends the impression that I’m a likeable person”). The social performance-avoidance goal subscale consists of eight items assessing goals of avoiding negative evaluations of one’s social attributes (e.g., “I always want to avoid negative evaluations of my personality”). Each item is rated on a 4-point Likert scale ranging from 1 (strongly disagree) to 4 (strongly agree), with higher scores indicating greater endorsement of each type of social goal. The SGOS has sufficient reliability and validity (Kuroda and Sakurai, 2001, 2003, 2011).

Because the present school-based survey included multiple measures and was administered to young adolescent participants within a limited time, it was important to minimize response fatigue and careless responding and thus maintain data quality. In addition, the survey needed to be completed within the available time. For these reasons, the total number of items was reduced to an appropriate level for this survey context. To preserve the content coverage and reliability of each subscale, we selected the items based on both psychometric and theoretical rationales. First, within each original subscale, we selected items with the highest factor loadings reported in the scale-development study (Kuroda and Sakurai, 2001). Second, we selected items based on their definitional relevance to the construct and their representativeness of the original subscale. Consequently, we adopted the seven social learning goal items, five social performance-approach goal items, and five social performance-avoidance goal items.

In this study, Cronbach’s *α* was 0.79 for the social learning goal subscale, 0.85 for the social performance-approach subscale, and 0.85 for the social performance-avoidance subscale. These internal consistency coefficients were comparable to those reported in the original scale-development study by Kuroda and Sakurai (2001), which were 0.87, 0.88, and 0.87, respectively.

#### 2.2.2 Coping with friend-related stress

Relationship-rebuilding and positive reappraisal coping strategies were assessed using the positive relationship-oriented strategies subscale (16 items) of the Interpersonal Stress-Coping Inventory (Kato, 2000). This subscale assesses individuals’ efforts to actively improve, maintain, or sustain friendships and engage in positive reappraisal. For the same methodological reasons described above, including the need to maintain data quality and to complete the survey within the available time, we used a shortened version of the subscale. To preserve the measurement quality of the shortened subscale, we selected items based on both psychometric and theoretical criteria: the highest factor loadings reported in Kato’s (2000) scale-development study, definitional relevance to the construct, and representativeness of the original subscale. Based on these criteria, we adopted six items. Sample items include “I tried to better understand the friend,” “I thought I learned something from this experience,” and “I actively tried to interact with that friend.”

Scales measuring relationship-withdrawal and relationship-changing coping had not been developed in Japan when this study was conducted. Therefore, our research team followed four steps to develop new scales. First, we clarified the conceptual definitions of the constructs, as described in the Introduction. Second, we collected or generated candidate items that reflected those definitions, drawing on both a relevant existing measure (the negative relationship-oriented strategies subscale of the Interpersonal Stress-Coping Inventory; Kato, 2000) and newly created items. Third, from the initial item pool, we selected the items that best represented each construct. Finally, the wording of each item was carefully adjusted to ensure that it could be easily understood by junior high school students.

The relationship-withdrawal coping scale comprises seven items (e.g., “I completely stopped interacting with my friends,” “I chose to be alone,” “I turned down invitations from friends to hang out, no matter who invited me,” and “I tried not to talk to people around me as much as possible”). The relationship-changing coping scale comprises eight items (e.g., “I looked for new friends who were a better match for me,” “I tried to get closer to my other friends,” “I tried to make new friends,” and “I joined a different group of peers”).

Participants were asked to rate the frequency of these behaviors and thoughts in response to friend-related stress that they had experienced over the past three months on a 4-point Likert scale ranging from 0 (not at all) to 3 (frequently). Higher scores indicate more frequent use of each coping strategy. Regarding internal consistency in the present sample, Cronbach’s *α* was 0.80 for the relationship-rebuilding and positive reappraisal subscale, 0.67 for the relationship-withdrawal subscale, and 0.76 for the relationship-changing subscale.

### 2.3 Procedure

When our study was conducted (2002), Institutional Review Boards had not yet been established in most faculties of psychology in Japan, including our own. Therefore, to ensure the meeting of the required ethical standards, we carefully reviewed the survey purpose, procedures, and questionnaire items, in consultation with fellow peer researchers and the participating school principal and teachers. The questionnaire was anonymous; therefore, students could not be identified from their responses.

The final consent to conduct this study and participation of the students was obtained from the school principal. Because the local legislation and institutional requirements did not require informed parental consent for anonymous low-risk school-based surveys at the time of data collection, approval for such surveys was, in practice, typically left to the discretion of school principals under ordinary school procedures in Japan.

The classroom teachers explained the purpose of the study and distributed questionnaire packets to the students. The students were assured of confidentiality and anonymity and agreed to participate in the study by voluntarily responding to the questionnaire.

### 2.4 Data analysis

We conducted a path analysis to evaluate the hypotheses. The parameters were determined using maximum likelihood estimation. Because our data had multivariate nonnormality, we employed a bootstrapping method (with 2,000 resamples) to estimate parameters (Byrne, 2010; Nevitt and Hancock, 2001). This approach is useful when applying maximum likelihood estimation to data that violate the assumption of multivariate normality, particularly for reducing bias in standard error estimates and yielding more accurate confidence intervals (Byrne, 2010; Nevitt and Hancock, 2001). AMOS software (version 19.0) was used to conduct the path analysis with the bootstrapping method, and SPSS (version 23.0) was used to compute the descriptive statistics, multivariate normality, and correlations.

## 3 Results

### 3.1 Descriptive statistics and intercorrelations of the measures

Table 1 presents the means, standard deviations, skewness, and kurtosis of the study measures. The means and standard deviations did not suggest marked floor or ceiling effects for any variable except relationship-withdrawal coping, which showed a relatively low mean score. Skewness values ranged from −0.49 to 0.80, and kurtosis values ranged from −0.75 to −0.09. All values were within the commonly used cutoffs of 2 for absolute skewness and 7 for absolute kurtosis (West et al., 1995), suggesting no substantial univariate nonnormality.

**Table 1:** Means, standard deviations, skewness, and kurtosis for variables.

Variable	*M*	*SD*	Skewness	Kurtosis
Social learning goals	21.81	4.22	−0.49	−0.41
Social performance-approach goals	14.82	3.64	−0.27	−0.75
Social performance-avoidance goals	12.94	3.75	−0.07	−0.32
Relationship-rebuilding and positive reappraisal coping	9.31	4.54	−0.06	−0.66
Relationship-changing coping	8.14	5.10	0.26	−0.65
Relationship-withdrawal coping	3.74	3.38	0.80	−0.09

Table 2 presents correlations among the study measures. Regarding the correlations directly relevant to the study hypotheses, social learning goals showed significant positive correlations with relationship-rebuilding and positive reappraisal coping (*r* = 0.57, *p* < 0.01), and social performance-approach goals showed significant positive correlations with relationship-changing coping (*r* = 0.39, *p* < 0.01), consistent with Hypotheses 1 and 3, respectively. Social performance-avoidance goals were not significantly correlated with relationship-withdrawal coping (*r* = 0.14, *p* = 0.11), which was not consistent with Hypothesis 2. However, because these zero-order correlations alone could not clarify the unique associations between each social goal and each coping strategy while controlling for the other social goals, the hypotheses were evaluated based on the path analysis reported below. Sex was significantly correlated with social learning goals, relationship-rebuilding and positive reappraisal coping, and relationship-changing coping, indicating that girls scored higher than boys on these measures.

**Table 2:** Correlations among study variables.

Variable	1	2	3	4	5	6
1. Social learning goals						
2. Social performance-approach goals	0.45**					
3. Social performance-avoidance goals	0.33**	0.59**				
4. Relationship-rebuilding and positive reappraisal coping	0.57**	0.45**	0.26**			
5. Relationship-changing coping	0.23**	0.39**	0.29**	0.36**		
6. Relationship-withdrawal coping	−0.03	−0.01	0.14	0.12	0.52**	
7. Sex	0.18*	0.08	−0.03	0.17*	0.17*	0.06

Sex was coded as 1 = boys, 2 = girls. * *p* < 0.05, ** *p* < 0.01 (two-tailed tests).

### 3.2 Test of hypotheses

Before testing the hypotheses, we assessed multicollinearity by examining variance inflation factors (VIFs). VIFs ranged from 1.26 to 1.73, well below the threshold of 10.00 (Kline, 2011), indicating no collinearity.

We also examined multivariate normality, which is an assumption for the maximum likelihood estimation used in the path analysis. Mardia’s tests of multivariate skewness and kurtosis were significant (skewness: *z* = 102.34, *p* < 0.01; kurtosis: *z* = 2.46, *p* < 0.05), indicating that the data departed from multivariate normality. Therefore, we used the bootstrap procedure with 2,000 resamples in the path analysis (Byrne, 2010; Nevitt and Hancock, 2001).

**Figure 1:**
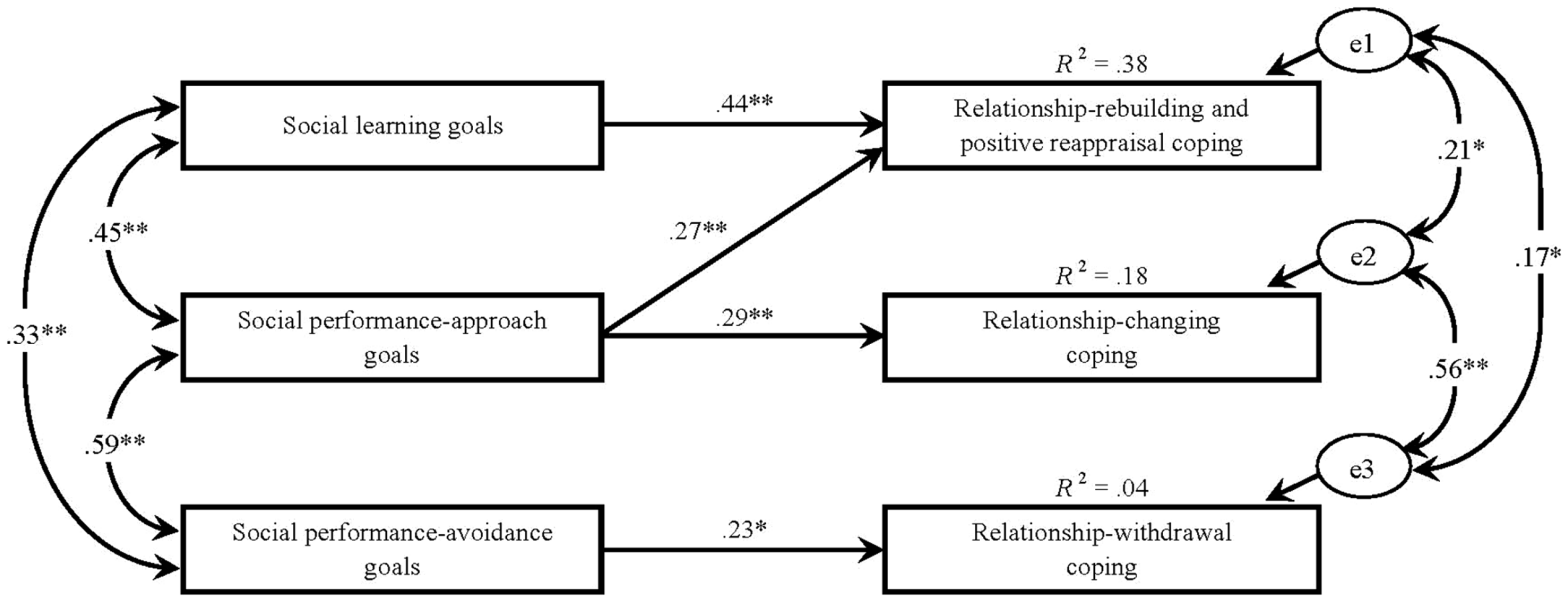
Results of the path analysis. Non-significant paths and covariances, as well as all paths and covariances involving sex (included as a control variable), are omitted for clarity. All path coefficients are standardized. *p < 0.05, **p < 0.01 (two-tailed).

Figure 1 shows the standardized path coefficients. All three hypothesized paths were significant and positive: from social learning goals to relationship-rebuilding and positive reappraisal coping (*β* = 0.44, bias-corrected 95% CI [0.28, 0.58]; Hypothesis 1 supported), from social performance-avoidance goals to relationship-withdrawal coping (*β* = 0.23, bias-corrected 95% CI [0.04, 0.41]; Hypothesis 2 supported), and from social performance-approach goals to relationship-changing coping (*β* = 0.29, bias-corrected 95% CI [0.05, 0.50]; Hypothesis 3 supported). A significant positive path from social performance-approach goals to relationship-rebuilding and positive reappraisal coping also emerged (*β* = 0.27, bias-corrected 95% CI [0.06, 0.51]), although this path was not hypothesized. The remaining path coefficients were not significant.

## 4 Discussion

This study examined whether the three social goals were differentially associated with engagement, disengagement, and mixed forms of coping in response to friend-related stress. Overall, the results supported our hypotheses.

### 4.1 Association between each goal and coping

Consistent with Hypothesis 1, social learning goals were positively associated with relationship-rebuilding and positive reappraisal coping. This association supports the theoretical view that social learning goals may energize and direct adolescents’ cognition and behavior toward social mastery and personal growth in the context of interpersonal stress (Dweck and Leggett, 1988). For adolescents who pursue these goals, experiencing stress in friendships may not conflict with, and may even support, their goal of growing through social experience. Thus, they may interpret these experiences as a challenge rather than a threat to the self (Lazarus and Folkman, 1984), thereby actively using engagement coping strategies.

In contrast, social performance-avoidance goals were positively associated with relationship-withdrawal coping, supporting Hypothesis 2. This finding supports the theoretical view that avoidance-oriented social performance goals may heighten adolescents’ sensitivity to negative evaluations and motivate them to disengage from potentially threatening social interactions (Kuroda and Sakurai, 2011; Shim and Ryan, 2019). In the context of friend-related stress, such sensitivity may not merely lead adolescents to avoid a specific friend but may promote broader withdrawal from friendships and peer relationships, thereby contributing to social isolation.

This withdrawal may reflect a strategic effort to minimize the risk of further negative evaluations. By retreating, adolescents may be able to shield their social self-worth. However, they may instead incur greater costs. Specifically, by isolating themselves from social relationships, they may feel lonely and depressed and, in the long term, may lose developmentally important opportunities to develop social relationships and cultivate social competence. Accordingly, relationship-withdrawal coping may be maladaptive.

Social performance-approach goals were positively associated with relationship-changing coping, consistent with Hypothesis 3. This finding supports the theoretical view that approach-oriented social performance goals may motivate adolescents to restore positive social evaluations after experiencing friend-related stress (Kuroda and Sakurai, 2011). For adolescents pursuing social performance-approach goals, negative feedback from a friend contradicts the positive feedback that they strive to gain. Such experiences may elicit frustration, which may lead them to avoid that friend (a disengagement strategy). However, because they focus on potential positive feedback, they may seek new friends who are likely to respond positively or strive to maintain other existing friendships (an accommodative engagement strategy). Thus, their coping pattern may contrast with that of adolescents pursuing social performance-avoidance goals.

The disengagement component of relationship-changing coping may be maladaptive because it does not address the source of stress. In contrast, the accommodative engagement component may be adaptive because it helps adolescents maintain social connections. This component may be particularly beneficial when primary control engagement coping is not helpful, such as when adolescents cannot directly resolve the source of friend-related stress (e.g., uncontrollable negative behaviors stemming from a friend’s temperament). A review by Clarke (2006) showed that the effectiveness of adolescents’ coping with interpersonal stress depends on the controllability of the stress, suggesting that active coping, such as primary control engagement coping, is helpful for controllable interpersonal stress but not for uncontrollable stress. In such cases, adolescents may need to adopt alternative strategies, such as seeking or maintaining other friendships, to maintain social connections.

Although not initially hypothesized, social performance-approach goals were significantly associated with relationship-rebuilding and positive reappraisal coping. Self-protective mechanisms may explain these associations. Specifically, adolescents with social performance-approach goals may engage in relationship-rebuilding coping to restore positive evaluations from their friends. This interpretation is consistent with prior research suggesting that individuals who pursue these goals strategically engage in socially desirable behaviors to gain positive feedback (Kuroda and Sakurai, 2003; Shim and Ryan, 2019). Furthermore, they may use positive reappraisal to shield themselves from the emotional impact of friend-related stress. Prior studies have demonstrated that adolescents with social demonstration-approach goals use nonchalance (Shin and Ryan, 2012) and defensive coping strategies (Michou et al., 2016), lending support to our interpretation.

These associations were all observed after controlling for sex, suggesting that the findings are not reducible to sex-related differences in social goals or coping. Instead, these associations may reflect fundamental motivational processes in which the defining characteristics of each social goal, such as the growth-oriented focus of social learning goals or the threat-sensitive nature of social performance-avoidance goals, may shape how adolescents cope with friend-related stress.

### 4.2 Comparisons between the roles of the three social goals in coping

Our findings suggest that the three social goals play contrasting roles in coping. The most critical difference lies between social learning goals and social performance-avoidance goals; the former motivates adolescents to repair friendships, whereas the latter leads them to withdraw from friendships. Theoretically, this contrast is rooted in the distinct fundamental foci of these two goals: a focus on growth and mastery versus a concern with negative evaluations of the social self.

Social performance-avoidance and social performance-approach goals have similar functions in that both motivate adolescents to avoid stressful friends. However, they differ in an important way: social performance-avoidance goals lead adolescents to withdraw completely from social relationships, whereas social performance-approach goals motivate them to seek other friendships. Thus, avoidance-oriented performance goals may increase adolescents’ risk of social isolation, whereas approach-oriented performance goals may help them maintain social connections. These findings suggest that even if adolescents focus on others’ evaluations, their coping strategies may differ depending on whether they strive to gain positive feedback or to avoid negative feedback.

The difference between social learning and social performance-approach goals may appear, at first glance, to be smaller than initially hypothesized because both goals are associated with relationship-rebuilding coping. However, it is important to consider that social learning goals are related *only* to relationship-rebuilding coping, whereas social performance-approach goals are related *not only* to relationship-rebuilding *but also* to relationship-changing coping. Given these findings, adolescents pursuing social learning goals may value their relationships with particular friends and thus strive to maintain or deepen those ties in the context of friend-related stress. In contrast, adolescents pursuing social performance-approach goals may show a less committed and more strategic orientation toward particular relationships, attempting to repair strained friendships while also distancing themselves from these relationships and seeking other friendships.

### 4.3 Theoretical and practical implications

Our findings extend those of prior studies (Michou et al., 2016; Shin and Ryan, 2012) by examining different forms of coping in a different stress context. By demonstrating the hypothesized associations, our study supports the predictive utility of social goal orientation theory across stress contexts and coping forms.

Furthermore, our study highlights the contrasting roles of the three social goals in coping, thereby supporting the discriminative utility of the trichotomous framework of social goal orientations. Notably, social performance-approach goals may play a more complex role in coping than previously recognized, involving both adaptive and maladaptive functions.

Our findings also have practical implications. Simultaneously cultivating interpersonal resilience and reducing vulnerability, rather than focusing on either alone, can enhance the effectiveness of prevention and intervention programs. This study proposes one such strategy: fostering social learning goals while addressing social performance-avoidance goals. This strategy shifts adolescents’ focus away from defending the social self—specifically, fearing and avoiding confirmation of their negative social attributes—toward developing the social self and meaningful relationships, thereby promoting constructive coping strategies and psychosocial adjustment. Targeting adolescents’ implicit theories of social traits (i.e., growth versus fixed mindsets) may facilitate this shift. According to social goal orientation theory, a growth mindset facilitates social learning goals, whereas a fixed mindset is more likely to promote performance-oriented goals (Dweck and Leggett, 1988).

### 4.4 Limitations and future directions

This study has several limitations. First, the cross-sectional and correlational design limits our ability to draw conclusions about causal relationships among the variables. Future studies should use longitudinal or experimental methodologies to clarify the directionality of these associations. The second limitation concerns the sample size and sampling method. Given the relatively small sample size of this study, caution is warranted when considering the stability of the parameter estimates. In addition, because participants were recruited from a single public junior high school, we should be cautious about generalizing the present findings to the broader adolescent population. Future research with larger and more diverse samples is required to replicate the present findings. The third limitation involves the short-form and newly developed scales employed. We did not conduct factor analyses of these scales because the current sample size (*N* = 137) was not sufficient to reliably test their factor structures. Furthermore, because some items were removed from validated subscales, the shortened scales may not have fully captured the breadth of the original constructs. Although the theoretically predicted findings provide some support for the nomological validity of these scales, independent studies are necessary to closely examine their factor structures and construct validity. Fourth, this study examined coping as a relatively stable tendency rather than as a situational or context-dependent process. According to contextual approaches to coping, people select and use coping strategies depending on situational demands and appraisals (Lazarus and Folkman, 1984; Moos and Holahan, 2003). In this process, contextual factors, such as the availability of resources including social support, play important roles. Prior research has also demonstrated that flexible selection and use of coping strategies are critical for mental health (Kato, 2012). How social goals function in these dynamic coping processes warrants further investigation. Fifth, social goal orientations are not the only factors that explain individual differences in coping, and other factors, such as attachment style and self-schema, are also important. Future research should integrate different theoretical approaches to examine and identify predictors of coping. Sixth, this study did not examine coping responses to major life events, such as bullying. The associations between the three types of social goals and coping with such events may differ from the patterns observed in this study. In particular, young adolescents with social performance goals may be more likely to adopt relationship-withdrawal coping in response to severe interpersonal stress, regardless of whether their goals are approach- or avoidance-oriented. How social goals relate to coping with stressors that differ in nature and severity should be investigated. Finally, because this study focused on the associations between social goals and coping strategies, adjustment indicators were not examined. Prior research has shown that engagement and disengagement strategies are linked to mental health outcomes and well-being (Compas et al., 2017; Zimmer-Gembeck and Skinner, 2016), suggesting a sequential pathway from social goals to coping and, in turn, to adjustment. Future studies should examine this pathway using a longitudinal design.

## 5 Conclusion

This study indicates that social learning, social performance-avoidance, and social performance-approach goals are associated with engagement, disengagement, and mixed forms of coping, respectively, suggesting that each goal plays a distinct role—whether adaptive, maladaptive, or both—in coping with friend-related stress. These findings support the predictive and discriminative utility of the trichotomous framework of social goal orientations for coping with interpersonal stress. Future research should extend the present findings by examining the longitudinal associations between social goal orientations, coping, and mental health using larger, more diverse samples and more established measures.

## Data Availability

The raw data supporting the conclusions of this article will be made available by the authors, without undue reservation.

## Footnotes

1 Social goals vary in type and level and are not limited to social learning and performance goals (see King and McInerney, 2019, for a comprehensive review). For example, the social goal content approach (e.g., Crick and Dodge, 1994; Renshaw and Asher, 1983; Wentzel, 2000) focuses on specific social goals, such as intimacy, domination, and responsibility, rather than on the broad, underlying goals posited in the social goal orientation approach. The social-academic approach (e.g., Dowson and McInerney, 2001; Urdan and Maehr, 1995) focuses on social reasons for engaging in academic work, such as social concern and social status, and therefore addresses both the social and academic domains, rather than the social domain alone, as in the social goal orientation and social goal content approaches. These different types and levels of social goals stem from a multiplicity of theoretical perspectives and are complementary rather than competing (Kiefer et al., 2013; King and McInerney, 2019; Ryan and Shim, 2006).

2 As discussed in the main text, Dweck’s theory (Dweck and Leggett, 1988; Erdley et al., 1997) posits that social goals are aimed at developing social attributes and social relationships (i.e., social learning goals) and validating social attributes (i.e., social performance goals). This theory further posits that these social goals are facilitated by implicit theories of social attributes (e.g., the malleable or fixed theory of personality). In these propositions, social attributes are specifically described as including personality traits, likability, and social competence (Dweck and Leggett, 1988; Erdley et al., 1997). Ryan and colleagues (e.g., Ryan et al., 2004; Ryan and Shim, 2006) adopted an achievement goal framework and focused on social competence among these social attributes, proposing the concept of social achievement goals. These goals comprise three types: social development goals, which aim to develop one’s social competence and social relationships; social demonstration-approach goals, which aim to gain positive judgments of one’s social competence from others; and social demonstration-avoid goals, which aim to avoid negative judgments of one’s social competence from others. Each goal corresponds to social learning, social performance-approach, and social performance-avoidance goals, respectively. In this study, we used the labels “social learning,” “social performance-approach,” and “social performance-avoidance” because we focused on broad social attributes, including personality traits and likability.
